# PACIFIC: a lightweight deep-learning classifier of SARS-CoV-2 and co-infecting RNA viruses

**DOI:** 10.1038/s41598-021-82043-4

**Published:** 2021-02-05

**Authors:** Pablo Acera Mateos, Renzo F. Balboa, Simon Easteal, Eduardo Eyras, Hardip R. Patel

**Affiliations:** 1 John Curtin School of Medical Research, Australian National University, Canberra, ACT 2600 Australia; 2 EMBL Australia Partner Laboratory Network at the Australian National University, Canberra, ACT 2600 Australia; 3 National Centre for Indigenous Genomics, Australian National University, Canberra, ACT 2600 Australia; 4 IMIM - Hospital del Mar Medical Research Institute, 08003 Barcelona, Spain; 5 Catalan Institution for Research and Advanced Studies, 08010 Barcelona, Spain

**Keywords:** Computational biology and bioinformatics, Genetics, Molecular biology, Microbiology, Clinical microbiology, Infectious-disease diagnostics, Pathogens, Virology

## Abstract

Viral co-infections occur in COVID-19 patients, potentially impacting disease progression and severity. However, there is currently no dedicated method to identify viral co-infections in patient RNA-seq data. We developed PACIFIC, a deep-learning algorithm that accurately detects SARS-CoV-2 and other common RNA respiratory viruses from RNA-seq data. Using in silico data, PACIFIC recovers the presence and relative concentrations of viruses with > 99% precision and recall. PACIFIC accurately detects SARS-CoV-2 and other viral infections in 63 independent in vitro cell culture and patient datasets. PACIFIC is an end-to-end tool that enables the systematic monitoring of viral infections in the current global pandemic.

## Introduction

Acute respiratory tract infections are the third largest global cause of death, infecting 545 million people and claiming 4 million lives every year^1–3^. RNA viruses such as influenza, parainfluenza virus, respiratory syncytial virus, metapneumovirus, rhinovirus, and coronavirus are amongst the top pathogens causing respiratory infections and disease^4,5^. Novel respiratory diseases, including coronaviruses, cross species boundaries repeatedly. Since December 2019, millions of people have been affected by COVID-19, an infectious zoonotic disease caused by severe acute respiratory syndrome coronavirus 2 (SARS-CoV-2, NCBI Taxonomy ID: 2697049). Novel zoonotic coronaviruses also caused the 2002–2003 outbreak of SARS-CoV respiratory disease with at least 8,098 known cases and the ongoing 2012–2020 outbreaks of Middle East respiratory syndrome coronavirus (MERS-CoV) with at least 2,519 known cases^5–8^. This recurrent emergence of respiratory viruses warrants increased surveillance and highlights the need for rapid, accurate, and timely diagnostic tests.

Diagnostic testing, treatment, and disease severity are complicated by the occurrence of respiratory co-infections. Up to 40% of individuals infected with respiratory viruses test positive for co-infections with up to three different pathogens^9,10^, and recent studies have reported that ~ 20% of SARS-CoV-2 positive individuals had a co-infection with other respiratory viruses^11^. Viral co-infections can alter the severity of disease and modify survival rates. While COVID-19 remains poorly understood, early studies indicate a potential for increased mortality associated with influenza co-infections^12^. Further studies are required to investigate the relationship between SARS-CoV-2 co-infections with prognosis and mortality rate^12,13^.

Current diagnostic tests for respiratory infections are often limited in their capacity to detect co-infections. The current standard of viral detection for COVID-19 is based on polymerase chain reaction (PCR) assays directed towards SARS-CoV-2^14^, which do not detect co-infecting viruses. Multiple virus identification in clinical settings is typically performed by using multiplexed PCR assays with primers specifically designed to target known respiratory pathogens^15^. However, this approach can generally only detect pathogens that are expected a priori because the range of pathogen detection is limited by the probe design. Additionally, these protocols must be updated as new species or strains are identified as clinically relevant.

High-throughput RNA sequencing (RNA-seq) provides an unbiased measurement of the RNA molecules present in a sample and can potentially enable the systematic detection of SARS-CoV-2 infections and co-infections. Multiple species are effectively detected in sequence data in the context of metagenomics studies^16^. Programs such as Kraken^17–19^ use k-mers to taxonomically classify sequence reads into species from metagenomic samples. However, these tools require large reference databases of species sequences to compare against, resulting in considerable storage and computing requirements. By comparison, machine learning based tools can extract the required features and encapsulate the necessary information for sequence classification in a computationally efficient way. This approach has been successfully used in the past to address sequence classification problems^20^. For example, DeepMicrobes^21^ uses deep learning for genus and species level classification of metagenomic DNA sequence reads from human gut bacteria. Similarly, ViraMiner^22^ uses a deep learning binary classifier to identify DNA viruses from human microbiome metagenomic reads. Despite these advances, there is currently no equivalent deep learning classifier suitable for the detection of SARS-CoV-2 and possible co-infections by RNA viruses.

To address this limitation, we have developed PACIFIC, a deep learning model to detect the presence of SARS-CoV-2 and other common respiratory RNA viruses in RNA-seq data from patient samples. PACIFIC discriminates reads into five distinct classes: SARS-CoV-2, influenza (representing H1N1, H2N2, H3N2, H5N1, H7N9, H9N2, and Influenza B), metapneumovirus, rhinoviruses (representing rhinovirus A, B and C) and other coronaviruses (representing alpha, beta, gamma, and other unclassified coronaviruses). PACIFIC achieves > 99% accuracy for virus detection, enabling the systematic identification of co-infections to improve clinical management and surveillance during the current pandemic.

## Results

### PACIFIC model

**Figure 1:**
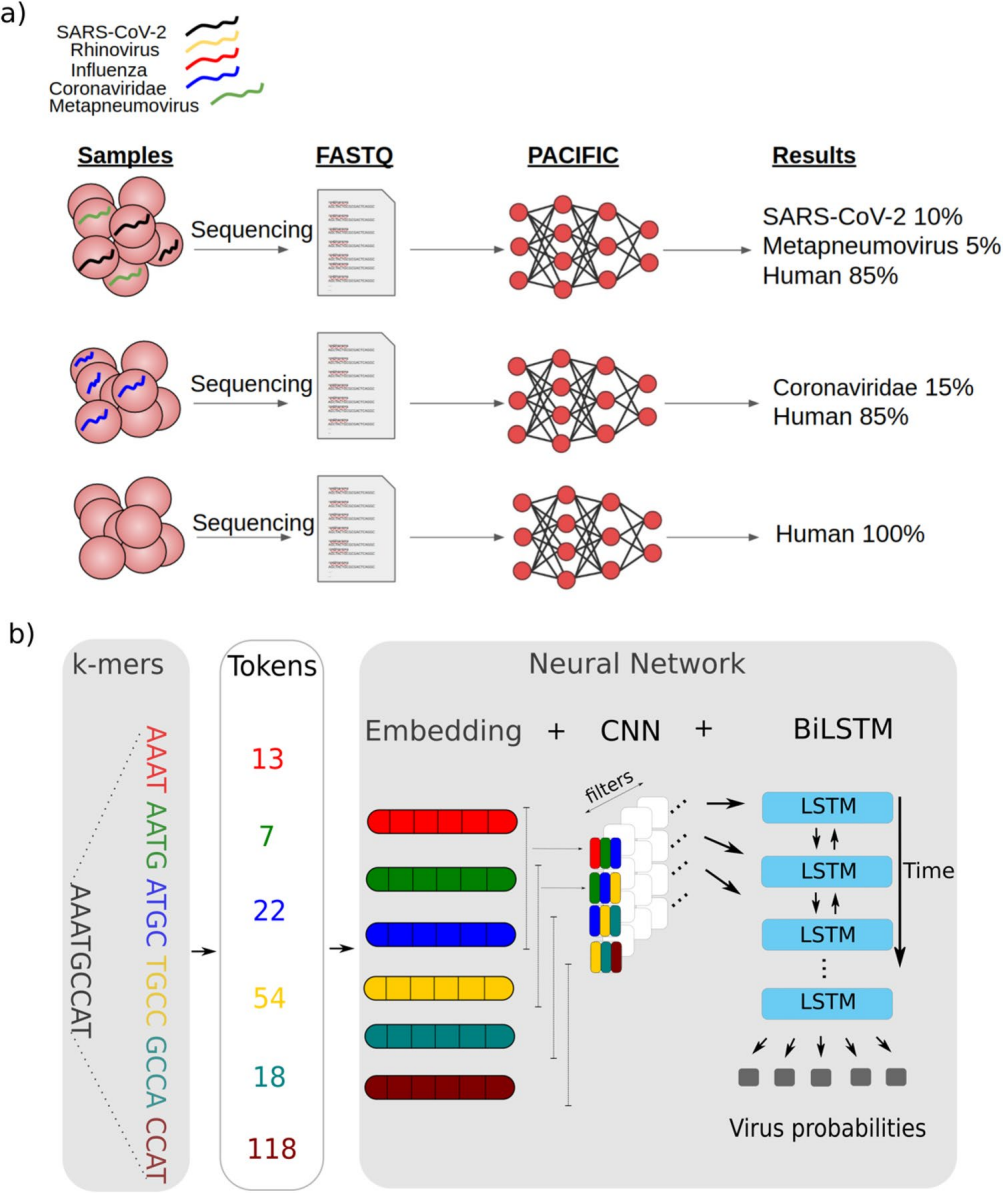
Overview of PACIFIC and its model architecture (**a**) PACIFIC uses FASTQ or FASTA files as inputs to make read-level predictions and to estimate the relative number of reads belonging to each RNA virus or human class. (**b**) PACIFIC deep neuronal network architecture, which includes embedding of k-mer tokens, convolutional neural network filtering to BiLSTM layers. The model is trained using in silico generated sequences from RNA virus genomes and the human transcriptome. CNN—convolutional neural network; BiLSTM—bi-directional long short-term memory.

PACIFIC is a deep learning method designed to classify RNA-seq reads into five distinct respiratory virus classes and a human class (Fig. 1a). The model architecture for PACIFIC is composed of an embedding layer, a convolutional neural network (CNN), and a bi-directional long short-term memory (BiLSTM) network that ends in a fully connected layer (Fig. 1b). One of the main advantages of deep neural networks compared to other machine learning models for sequence classification is the ability to extract relevant complex classification features from DNA or RNA sequences without having to explicitly define them a priori. However, the strategy to encode nucleotide sequences must be carefully considered, as it can dramatically affect the performance of the classifier^23^. The embedding layer improves the performance of the model relative to other encoding approaches^24^. PACIFIC first converts nucleotide sequences into k-mers, assigns these to numerical tokens and converts the tokens into dense representations using a continuous vector space.

The use of a convolutional neural network adds several advantageous properties to the model. One advantage is location invariance^25^, which allows the model to identify combinations of features with predictive value regardless of their relative position along the sequence. In addition, each filter used in the convolution layers can capture the predictive value of specific regions or combinations of k-mers.

After the convolution layers, PACIFIC uses a pooling layer to decrease the dimensionality of the feature space while maintaining essential information. PACIFIC uses a BiLSTM to model long-range dependencies in nucleotides, which provides the capacity to incorporate complex relationships in the input sequence that are sometimes ignored by single LSTMs^26^. PACIFIC then implements a dense layer to estimate posterior probabilities for each of the five classes considered: Coronaviridae, Influenza, Metapneumovirus, Rhinovirus, and SARS-CoV-2. We included a sixth class in the model (Human) to classify RNA-seq reads derived from the human host. Finally, PACIFIC predicts the forward and reverse-complement of each read, and only assigns the read to a particular class if the posterior probability of the assigned class is maximal for that class for the forward and reverse-complement reads, and its value is ≥ 0.95.

### Properties of PACIFIC training data

PACIFIC was trained using 7.9 million 150nt long random fragments from 362 viral genome assemblies belonging to one of five viral classes (SARS-CoV-2, Influenza, Metapneumovirus, Rhinovirus and Coronaviridae) and the human transcriptome (Supplementary Table S1) (“Methods”). In silico fragments from both strands were generated without errors to accommodate paired-end sequence reads and to retain the natural variation among genomes within each class. We used 90% of the data for training and 10% for tuning the hyperparameters and network architecture.

The choice of virus classes was based on the following considerations. First, we wanted PACIFIC to accurately detect SARS-CoV-2 as an independent class and to discriminate it from other coronaviruses. Second, we chose viruses that have been recently reported to co-infect patients with SARS-CoV-2^11^. Third, we included only viruses for which humans are identified as a host species in the NCBI Taxonomy database^27^. Fourth, as the majority of reads in a sample are expected to be derived from human RNAs, we included an independent human class representing the human transcriptome to avoid misclassifying human reads into one of the viral classes.

### K-mer length selection and sequence divergence

As input reads are divided into k-mers within the model, we investigated appropriate virus and human k-mer properties. A k-mer length of 9 has previously been identified as optimal for the phylogenetic separation of viral genomes^28^. However, 9-mer profiles of SARS-CoV-2 and the human transcriptome have not been previously explored. We computed all-vs-all Jensen-Shannon divergence (JSD) scores using 9-mers to confirm that k = 9 effectively distinguishes between the six PACIFIC classes. JSD, which is a symmetric measure of the (dis)similarity between k-mer probability distributions^29^, range between 0 for identical sequences and 1 for two sequences that do not share any k-mer. Overall, inter-class JSD values were higher compared to the intra-class JSD values for 9-mers, which confirm that 9-mers effectively separate sequences belonging to different viral classes and human transcripts (Supplementary Fig. S1). Specifically, the average JSD between the SARS-CoV-2 class and the Coronaviridae class was 0.79, which was greater than 0.7 intra-class JSD for the Coronaviridae and 0.001 for the SARS-CoV-2 class, thus indicating sufficient divergence for their separation into distinct classes. Given these results, we decided to encode input sequences as 9-mers with a stride of 1.

### PACIFIC testing shows high precision and recall for simulated data

Performance metrics (false positive rates (FPRs), false negative rates (FNRs), precision, recall, and accuracy, Eqs. (3–7)) were calculated using in silico generated reads that modelled sequencing-induced substitution and indel errors in sequence mixtures with known class labels. We generated 100 independent datasets of 150nt single end reads with Illumina HiSeq 2500 errors using ART^30^. Each dataset contained ~ 700,000 reads and was comprised of approximately 100,000 reads from each of the 6 classes in the PACIFIC model, plus ~ 100,000 reads from unrelated viral genomes (viral genomes that did not belong to the five classes that PACIFIC tests for; “Methods”). PACIFIC took an average of 108 s to process 100,000 reads on a machine with an AMD Ryzen Threadripper 2950 × 16-core processor, NVIDIA GeForce RTX2080Ti GPU, and 125.7 GiB RAM running Ubuntu 18.04.2 LTS.

### Single vs double predictions

**Figure 2:**
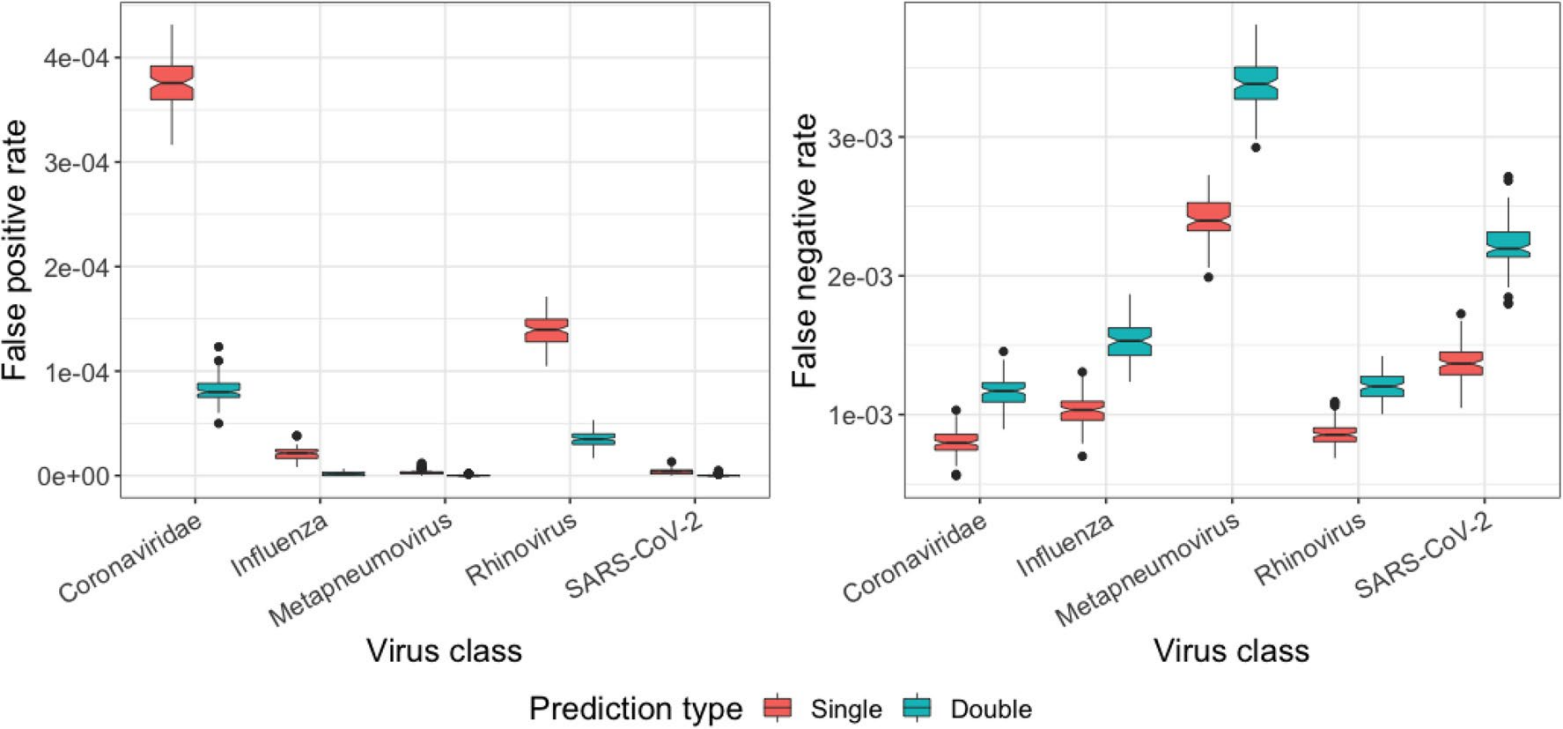
**Comparison of false positive and false negative rates between** ***single*** **and** ***double predictions***. *Single prediction* (red) results in relatively higher false positives and lower false negatives compared to *double prediction* (green) where predictions are made on both the positive strand of a sequence read and its reverse complement.

First, we compared the performance metrics of predictions using only direct reads (*single prediction*) and predictions using both the direct reads and their reverse complements (*double prediction*) (Fig. 2). In *single prediction*, a read was assigned the class label with the highest posterior probability for the direct read if the posterior probability for a class was ≥ 0.95. For *double prediction*, a read was predicted to belong to a given class if the posterior probability of both the direct read and its reverse-complement was ≥ 0.95 and predictions agreed on the same class. Across 100 datasets average FNR for *double prediction* increased relative to *single prediction* by 1.45 × for Coronaviridae, 1.49 × for Influenza, 1.41 × for Metapneumovirus, 1.40 × for Rhinovirus and 1.62 × for SARS-CoV-2 (Fig. 2). In contrast, we observed lower FPR for *double prediction*. The average FPR in the 100 datasets decreased by 4.60 × for Coronaviridae, 11.02 × for Influenza, 16.55 × for Metapneumovirus, 3.92 × for Rhinovirus and 16.47 × for SARS-CoV-2 class in *double prediction* relative to *single prediction* (Fig. 2). We therefore observe that the average precision for all viral classes increased substantially, outweighing the small decrease in average recall. Based on these observations, *double prediction* was implemented as the standard classification approach in PACIFIC as it reduced FPR by a higher margin.

Overall, PACIFIC achieved high precision (average ≥ 0.9995), recall (average ≥ 0.9966) and accuracy (average ≥ 0.9995) for each of the virus classes (Table 1). For the human class, the average precision was lower at 0.50140 compared to the viral classes. This lower precision is explained by a large number of reads (> 99%) from unrelated viral genomes being assigned to the human class. As a result, sequences that do not belong to any of the viruses in the model are unlikely to be mislabelled as one of the virus classes.

**Table 1:** Average of performance metrics for each class in 100 independent simulated datasets.

Class	FNR (± 95% CI)	FPR (± 95% CI)	Precision (± 95% CI)	Recall (± 95% CI)	Accuracy (± 95% CI)
*Coronaviridae*	0.116% (2.14e−05)	0.008% (2.38e−06)	> 0.999 (1.41e−05)	0.999 (2.14e−05)	> 0.999 (3.67e−06)
*Influenza*	0.153% (2.65e−05)	< 0.001% (3.18e−07)	> 0.999 (1.95e−06)	0.999 (2.65e−05)	> 0.999 (3.80e−06)
*Metapneumovirus*	0.340% (3.62e−05)	< 0.001% (1.04e−07)	> 0.999 (6.23e−07)	0.997 (3.62e−05)	> 0.999 (5.20e−06)
*Rhinovirus*	0.121% (1.90e−05)	0.004% (1.41e−06)	> 0.999 (8.44e−06)	0.999 (1.90e−05)	> 0.999 (2.79e−06)
*SARS*-*CoV*-*2*	0.222% (3.27e−05)	< 0.001% (1.43e−07)	> 0.999 (8.63e−07)	0.998 (3.27e−05)	> 0.999 (4.64e−06)
*Human*	0.037% (1.32e−05)	0.166% (1.03e−05)	0.501 (1.45e−05)	> 0.999 (1.32e−05)	0.858 (8.94e−06)

*CI* confidence interval, *FPR* False positive rate, *FNR* False negative rate.

### Effect of mismatches and natural variation on predictions

**Figure 3:**
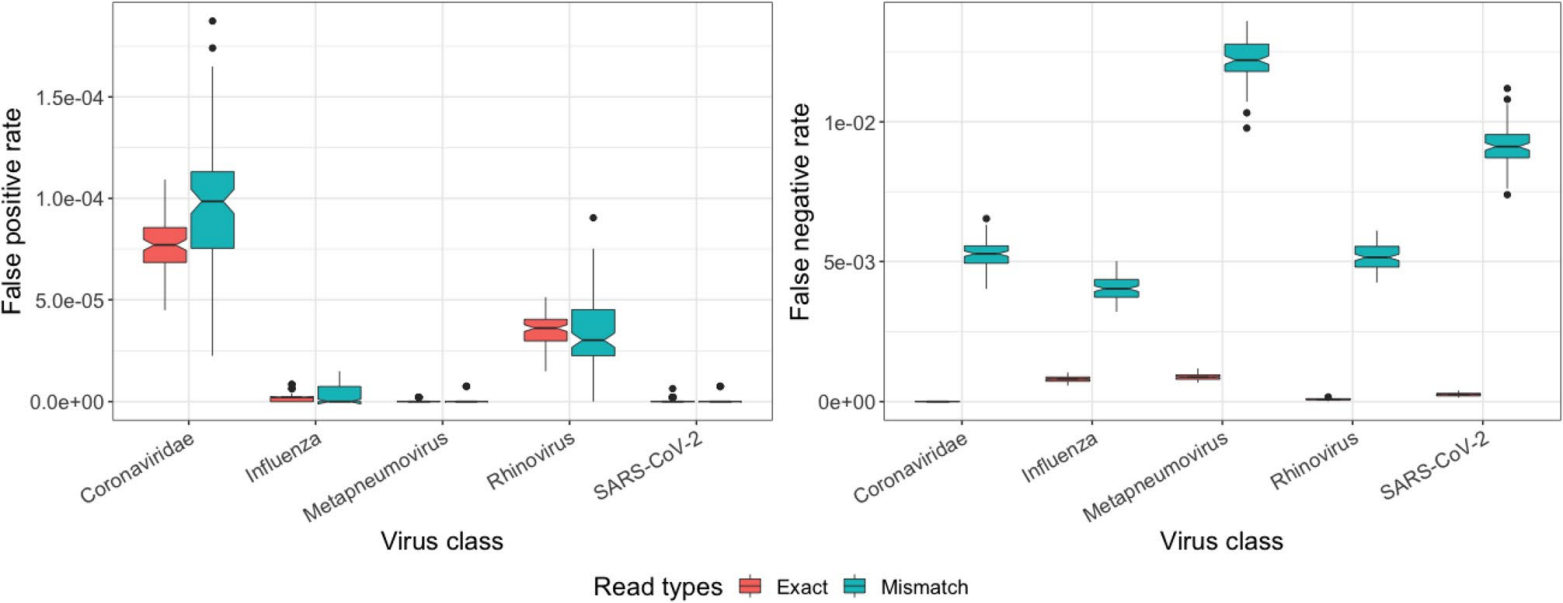
False positive (left panel) and false negative (right panel) rates for reads identical to the corresponding reference genome (Exact, red), and for reads with mismatches with respect to their reference genome (Mismatch, green).

Using the same in silico datasets, we then assessed the effect of mismatches on FNR and FPR. All 100 controlled datasets contained ~ 22% of reads with substitutions and indel errors relative to the reference genomes. FNRs were higher for mismatch-containing reads relative to exact reads for all viral classes. For the Coronaviridae class, all false negative reads contained mismatches. Similarly, FNRs increased 5 to 64-fold in the other four viral classes (Fig. 3). FPRs increased 0.98 to twofold for mismatch-containing reads for all classes. These results suggest that FPRs are relatively less affected by the presence of mismatches compared to FNRs for viral classes. Despite relative differences of FPR and FNR in mismatch-containing reads compared to exact reads, PACIFIC achieved high precision (0.999), high recall (0.998) and high accuracy (0.998) for mismatch-containing reads for all five viral classes (Supplementary Table S2).

To assess the impact of natural variation observed during the current pandemic on the sensitivity of predicting SARS-CoV-2, we performed 200 simulated experiments using more than 20,000 SARS-CoV-2 sequences separated into a low and a high divergence set (“Methods”). The low-divergence set on average contained 7.3 mismatches (standard error: 0.07) per SARS-CoV-2 assembly relative to the reference genome. In contrast, the high-divergence set contained on average 16.4 mismatches (standard error: 0.05) relative to the reference genome. An average of 0.05 reads out of 100,000 SARS-CoV-2 reads were falsely assigned to the Coronaviridae class in the low-divergence set. These false-negative assignments to the Coronaviridae class for SARS-CoV-2 increases negligibly to an average of 0.26 reads per 100,000 reads for the high-divergence set. There were no false-negative assignments to any other viral class in either set. In addition, true positive SARS-CoV-2 assignments were similar between both sets (0.994 and 0.995 for low-divergence and high-divergence sets respectively). These results show that PACIFIC is robust to the natural sequence variation observed in the SARS-CoV-2 strains across the world.

### Investigating predicted and actual labels

**Figure 4:**
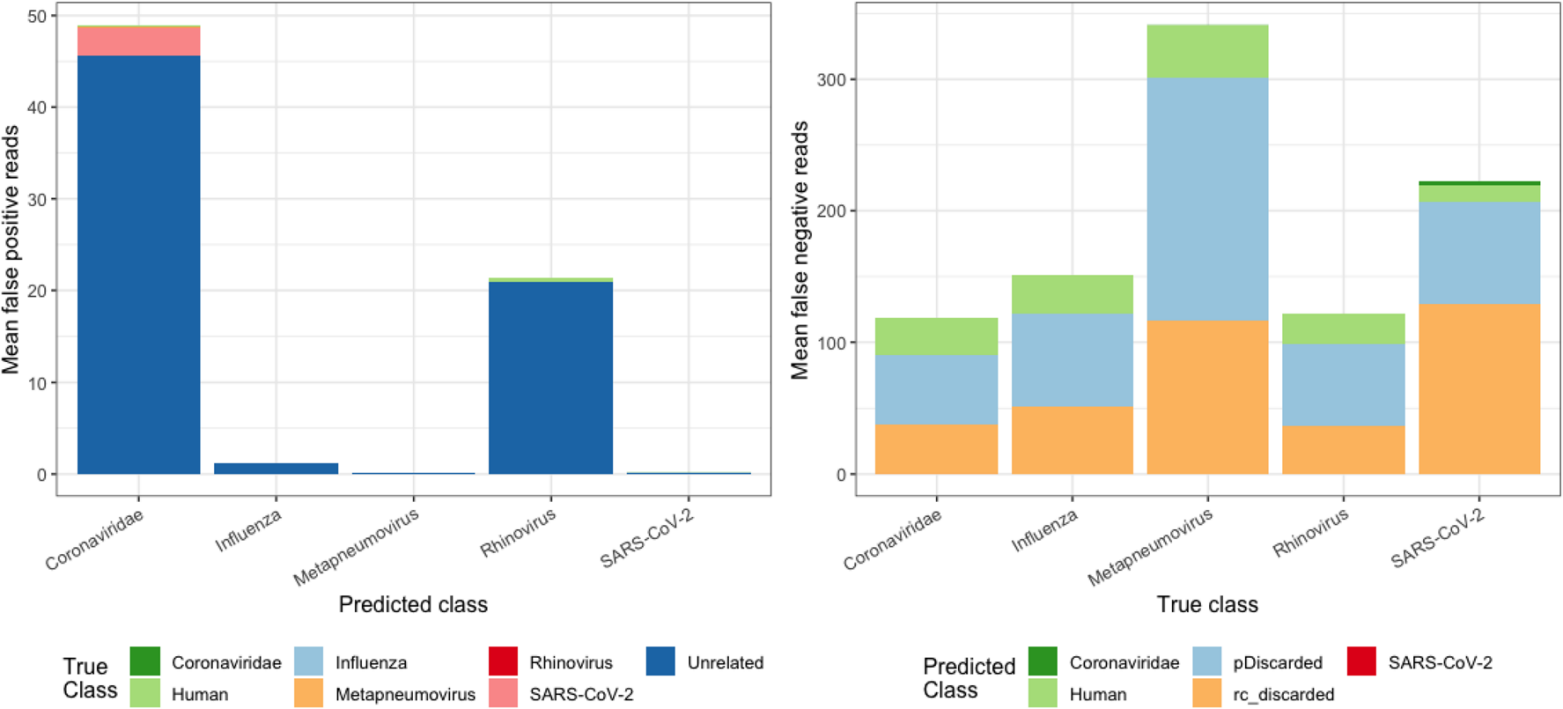
False positive and false negative assignments for 100 independently simulated datasets. Left panel—Average number of false positives (y axis). True labels are indicated by colour for each class and predicted labels are given in the x-axis. Right panel—Average number of false negatives (y axis). Predicted labels are indicated by colour for each class and true labels as indicated in the x-axis. pDiscarded—reads that do not reach the 0.95 posterior probability cut-off; rc_discarded—reads that were discordantly predicted using *double prediction*.

Reads derived from unrelated virus genomes contributed most of the false positives for all predicted classes (Fig. 4). Of note, there was negligible cross contamination between viral class labels. The largest inter-viral class misclassification was from SARS-CoV-2 to Coronaviridae, where out of 100,000 SARS-CoV-2 reads, ~ 2.9 were misclassified as Coronaviridae. This was expected as SARS-CoV-2 is a species within Coronaviridae genus. Between other viral classes, only 0–0.2 per 100,000 reads were misclassified. Reads were discarded because of the class mismatch for the read and its reverse complement in *double prediction* (rc_discarded in Fig. 4), or because they did not meet the minimum 0.95 posterior probability criteria (pDiscarded in Fig. 4) contributed maximally to false negatives. Taken together, our results demonstrate that PACIFIC is highly specific and sensitive for all five viral classes, with negligible false positive and false negative rates.

### Establishing virus detection thresholds

In the previous section, we showed that PACIFIC displayed low FPR in balanced datasets with similar proportions of reads from each class. However, incorrect predictions about the presence or absence of a virus in a sample could lead to misguided follow-ups and the unnecessary use of valuable clinical resources. To better assess the real-world performance of PACIFIC, we estimated the minimum proportion of sequence reads from a specific viral class that is required to confidently predict the presence of that virus in a sample.

In practice, RNA-seq data will be unbalanced, with only a small, variable proportion of viral reads combined with a majority of reads that originate from the human transcriptome. To assess the effect of this imbalance on false positive rates, we simulated 500 independent datasets (100 for each class), each containing 500,000 150nt reads, using ART^30^ with Illumina HiSeq 2500 error profiles. Each dataset contained variable proportions of simulated reads for 4 of the 5 viral classes, plus the Human class, which includes human and unrelated viral genomes. One of the five viral classes was intentionally excluded, and the excluded class was considered to be the test class. All reads assigned to this test class were counted as false positives for the remaining 4 classes. PACIFIC achieved similar average FPRs to the benchmarking experiments using balanced datasets (Table 2).

**Table 2:** Average false positive reads across 100 experiments for each viral class.

Class	FP/1 M reads (Balanced)	FP/1 M reads (Unbalanced)	FP% threshold^a^
Coronaviridae	81.6	66.6	0.0405
Influenza	1.9	6	0.000807
Metapneumovirus	2	1	0.000154
Rhinovirus	35.5	53.9	0.0418
SARS-CoV-2	2	1	0.000213

FP = False positive, 1 M = 1 million, Balanced = 100 experiments with equal proportion of reads from each class, Unbalanced = Variable proportion of reads from all classes and no reads from the test class.

^a^These thresholds represent 0.99 quantile for FP% for that class.

Assessment of the distribution of FPRs for each viral class and skewness-kurtosis plots indicated that the percentage of false positives observed in unbalanced datasets followed a Beta distribution. Therefore, we used moment matching to estimate the shape parameters for the quantile function of the Beta distribution and determined the numeric threshold above which 99% of false positive samples were excluded. Table 2 shows detection thresholds for each viral class above which samples are classified as positive for the viral class. For example, a sample is classified as positive for Coronaviridae if > 0.0405% reads are labelled as Coronaviridae using PACIFIC (Table 2).

### PACIFIC accurately detects viruses in human RNA-seq samples

Next, we assessed PACIFIC’s performance in classifying viral reads in RNA-seq data derived from human biological samples and compared its output with alignment-based (BWA-MEM) and k-mer based (Kraken2) approaches. To reduce bias in the comparisons, we built the BWA-MEM index and Kraken2 database using the same virus genome assemblies and the human transcriptome that were used for PACIFIC training (Supplementary Table S1). For all three methods, we used the thresholds established in the previous section (Table 2) to determine the presence of a virus in a sample. All three methods were applied to 63 human RNA-seq datasets from independent research studies, five of which are known to contain SARS-CoV-2^31,32^. Four datasets were derived from primary human lung epithelium cells (NHBE) infected with SARS-CoV-2 in vitro (NCBI SRA accession: SRX7990869)^31^. The other infected dataset was obtained from a patient bronchoalveolar lavage fluid sample (NCBI SRA accession: SRR10971381) that was positive for SARS-CoV-2^32^. In addition, we analysed RNA-seq datasets for 48 airway epithelial cell samples from the GALA II cohort study, of which 22 were reported to contain respiratory infection viruses^33,34^, and 10 were reportedly devoid of infections by viruses in the virus classes used here, as indicated by the sample metadata and the corresponding publications (Supplementary Table S3).

**Figure 5:**
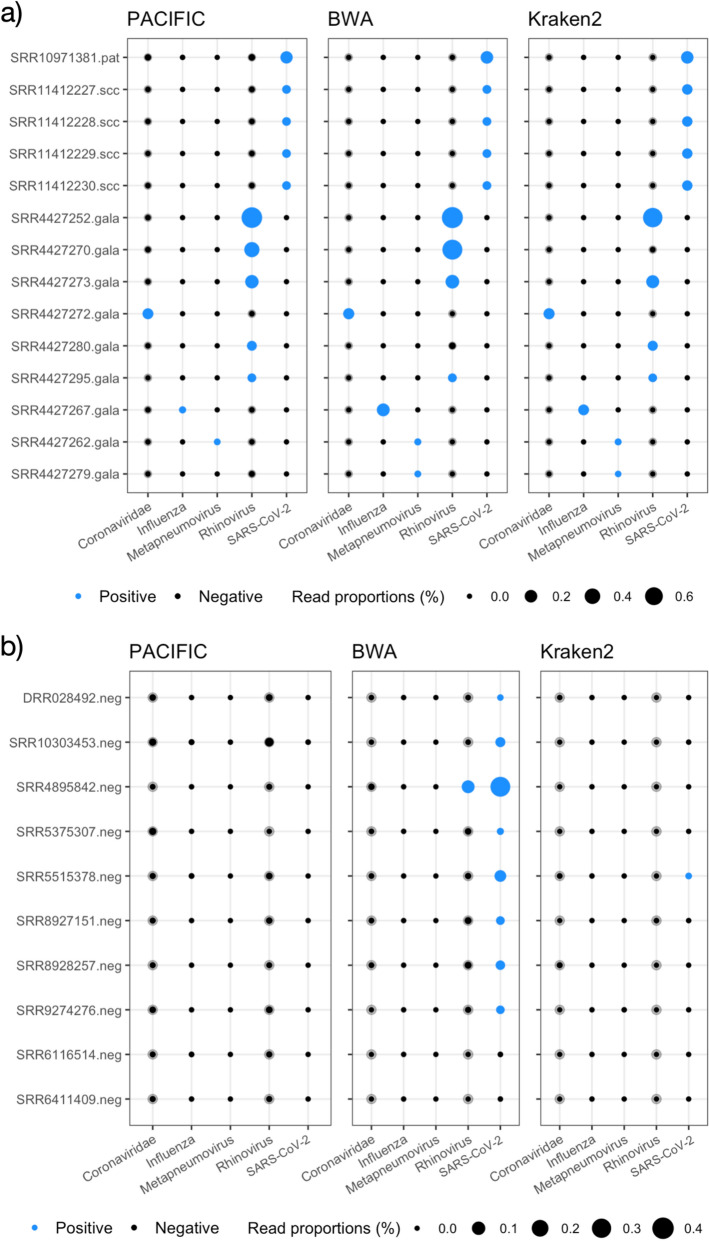
PACIFIC, BWA-MEM and Kraken2 virus predictions in RNA-seq data. Transparent grey filled circles represent detection thresholds for each class, overlaid with black filled circles representing the percentage of predicted reads using PACIFIC (left panel), BWA-MEM (centre panel) and Kraken2 (right panel). Circles are filled blue when the percentage of reads for a class are above detection thresholds described in Table 2. RNA-seq samples (y-axis) are labelled with NCBI SRA run accessions and abbreviations for sample type. (**a**) RNA-seq samples predicted to be positive for at least one viral class by PACIFIC, BWA-MEM, or Kraken2. Samples include a SARS-CoV-2-infected human patient bronchoalveolar lavage fluid sample (.pat), four in vitro SARS-CoV-2-infected NHBE cell lines (.scc) and 9 samples from the GALA II cohort (.gala). (**b**) Human RNA-seq samples without expected viral infections (.neg; n = 10). Samples were selected from the NCBI SRA database with no recorded evidence of infection.

For the five samples that were positive for SARS-CoV-2, PACIFIC assigned 0.047%-0.048% of all reads to the SARS-CoV-2 class for the in vitro infected cells and 0.19% of all reads in the patient sample. For all five samples the proportion of assigned reads is above the detection threshold for SARS-CoV-2 class (> 0.000213%) (Fig. 5). BWA-MEM and Kraken2 also successfully identified SARS-CoV-2 reads above the detection threshold in these five samples (Fig. 5a). Thus, all three methods accurately predicted the presence of SARS-CoV-2. In addition, all methods assigned fewer reads to the other virus classes than their respective detection thresholds, indicating the absence of other virus classes in these samples.

We subsequently tested the 48 samples from the GALA II cohort^33,34^. Of these, 22 were reported to contain between 4 and 164,870 reads from respiratory viruses (human rhinovirus, respiratory syncytial virus, human metapneumovirus or human parainfluenza viruses I, II and III). PACIFIC, BWA-MEM and Kraken2 identified 9 samples as positive for one of the five virus classes considered, and 39 samples as negative for the same viral classes (Fig. 5a, Supplementary Fig. S2, Supplementary Table S3). The discrepancy between these results and the original study^34^ could be partly explained by the exclusion of the Respiratory Syncytial Virus class in our analyses. However, further verifications could not be performed because sample labels provided in the manuscript were mismatched with the submitted sequence data (see correction^35^).

From the 9 positive samples, six were labelled positive for the same virus class by all three methods: three samples were positive for Rhinovirus, and one for Coronaviridae, Influenza, and Metapneumovirus, respectively (Fig. 5a). In contrast, the other three samples were discordantly labelled by one of the three methods. SRR4427279 was classified as positive for Metapneumovirus by BWA-MEM and Kraken2 but not by PACIFIC. BWA-MEM and Kraken2 collectively assigned 28 reads to the Metapneumovirus class as compared with 0 reads by PACIFIC. To investigate the origin of these 28 reads, we used BLASTN searches against the NCBI nucleotide (*nt*) database encompassing sequences from all domains of life and extracted the best hit for each read (“Methods”). All 28 reads had their best hits to human respiratory syncytial virus A sequences (E-values ≤ 4.07e-68; bit-scores ≥ 267). Furthermore, metapneumovirus was not identified in any of the top 10 significant hits (Supplementary Information: *Extension of BLAST analysis*). Another discordant sample (SRR4427270) was classified as positive for the Rhinovirus class by PACIFIC (1,065 reads) and BWA-MEM (2,338 reads) but not by Kraken2 (3 reads). BLASTN searches showed that best hits (3,145/3,150 total BLAST alignments) were sequences from one of: Enterovirus C105; Enterovirus C; or Human enterovirus C105 (E-values ≤ 9.46e-41, bit-scores ≥ 178). Rhinoviruses and other enteroviruses are taxonomically part of the *Enterovirus* genus^36,37^. The final discordant sample (SRR4427280) was labelled positive for the Rhinovirus class by PACIFIC (355 reads) and Kraken2 (387 reads) but not by BWA-MEM (29 reads) using our thresholds (Fig. 5a). BLASTN searches revealed that the majority of reads (385/390) labelled by either PACIFIC or Kraken2 had their best hits to Rhinovirus C (E-values ≤ 4.47e−53; bit-scores ≥ 219). BWA-MEM therefore failed to assign most Rhinovirus reads correctly classified by the other two methods.

In addition to the 53 samples tested above, we analysed 10 publicly available human RNA-seq datasets with no record of viral infection. The samples are unlikely to be infected with SARS-CoV-2 as they were registered in the NCBI SRA database on or before 17 October 2019 (Supplementary Table S3). Although two samples (SRR8927151 and SRR8928257) were published in February 2020 (Supplementary Table S3)^38^, the NCBI BioProjects for these samples were registered on 18 April 2019.

PACIFIC accurately predicted all 10 samples as negative for viral infections using our detection thresholds. In contrast, Kraken2 assigned one sample (SRR5515378) as positive for SARS-CoV-2 with 256 reads assigned (Fig. 5b). Further verification with BLASTN searches confirmed that 242 out of the 256 reads mislabelled by Kraken2 aligned to the *Mycoplasma* bacterial genus (E-values ≤ 2.76e−23, bit-scores ≥ 121), indicating false positive assignments by Kraken2 for these reads.

BWA-MEM performed relatively worse for these 10 datasets, with 8 samples classified as positive for SARS-CoV-2 (Fig. 5b). In these 8 samples, between 86 and 6,901 reads (total = 15,395 reads) were classified as SARS-CoV-2. BLASTN searches identified *Homo sapiens* as the best hit (E-values ≤ 5.78e−07, bit-scores ≥ 65.8) for 4352 (28%) of these reads. Further examination revealed that 53% of all 9-mers in these reads were poly-A or poly-T derived, suggesting low-complexity sequences. In addition, SRR4895842 was assigned as co-positive for both the Rhinovirus and SARS-CoV-2 classes by BWA-MEM (Fig. 5b). BLASTN searches of 406 reads assigned to Rhinovirus within this sample revealed that 303 reads had best hits to *Homo sapiens,* and 48 reads had their best hits to *Pan paniscus* (E-value ≤ 6.06e−10; bit-scores ≥ 76.8). These reads had 19% of their 9-mers derived from poly-A or poly-T sequences, suggesting low-complexity sequences.

Overall, these results show that PACIFIC accurately identifies viral reads and that our detection thresholds identify the presence or absence of viral classes in RNA-seq data from biological samples with fewer false positives and false negatives than other methods currently in use.

## Discussion

We have developed PACIFIC, a deep learning-based tool for the detection of SARS-CoV-2 and other common respiratory viruses from RNA-seq data. To the best of our knowledge, PACIFIC is the first deep learning model that detects SARS-CoV-2 and other RNA virus groups from short-read sequence data with > 0.99 precision, recall and accuracy. A recent analysis of 4,909 scientific articles identified 47 models for detecting COVID-19, 34 of which were based on medical images^39^. This study concluded that these predictive models were, in general, poorly described and contained multiple biases, likely resulting in unreliable predictions when applied in practice. We addressed these limitations by validating the performance of PACIFIC using diverse, independently simulated datasets that reflect realistic scenarios. We also show that PACIFIC performs well when applied to 63 RNA-seq datasets derived from infected cell cultures and patient samples, indicating its utility in clinical settings.

In 2013, the World Health Organisation launched the Battle against Respiratory Viruses (BRaVe) initiative, which identified six research strategies to tackle and mitigate risks of death due to respiratory tract infections. One of the proposed strategies was to “improve severe acute respiratory infection diagnosis and diagnostic tests amongst others”^40^. High-throughput sequencing has great potential in this context. The SARS-CoV-2 pandemic provides new impetus to evaluating its diagnostic application, thereby contributing to the BRaVe initiative’s goals^41,42^.

A comprehensive study using multiplex RT-PCR and a sequencing-based metagenomic approach revealed that RNA-seq has sufficient sensitivity and specificity to be applicable in the clinic for respiratory viruses^42^. However, the need for complex analytical workflows can limit the use of RNA-seq in diagnostic settings^34,42^. A typical workflow for virus detection in high-throughput sequencing data involves quality assessment and filtering of raw data, removal of host sequences, de novo assembly of remaining reads, and alignment and annotation of the assembled contigs^43^. Implementation of these workflows require expert knowledge of bioinformatics software and databases and dedicated computing facilities. PACIFIC overcomes these limitations by modelling the differences in k-mer content of respiratory viruses and human sequences in a model that is efficient in compute and storage requirements, easy to use, and therefore applicable in contexts with limited resources. Specifically, we have designed PACIFIC to be run as a single command using raw RNA-seq data as the only required input to obtain quantitative predictions about viral classes within a sample.

The higher costs of sequencing compared to PCR-based experiments can be reduced by multiplexing, block-testing or pooling strategies^44^, to enable unbiased cost-effective testing. For example, sequencing with Illumina platforms can be done with 96 samples per lane using multiplexing, reducing the sequencing cost per sample. In this scenario, the number of reads obtained per sample could be approximately 200,000 or higher. We have demonstrated the accuracy of PACIFIC in a variety of sample sizes, which suggests the potential value of this approach.

Identification of virus classes from sequence data is complicated by the high rate of natural sequence variation for RNA viruses^45,46^, experimental errors and artefacts in sequence data, and the presence of low-complexity A-rich sequences common to the host transcriptome. We showed that 22% of reads containing mismatches and indel errors were correctly assigned to a virus class by PACIFIC with negligible loss in sensitivity at a sample level (Fig. 3). In addition, PACIFIC accurately classifies SARS-CoV-2-derived reads despite natural sequence variation observed in publicly available data, without significant loss in sensitivity. We show that PACIFIC can be easily extended using the transfer learning approach to incorporate new classes and sequence diversity as new viral mutations emerge (“Methods”; Supplementary File 2).

PACIFIC has been developed specifically to identify viral classes reported to be co-infecting patients with SARS-CoV-2^12^. Samples containing other viruses and bacterial infections may require additional analysis. Future versions of PACIFIC could include the classification of a broader range of virus and bacterial classes at a species level, accommodate variable input read lengths to increase its utility in other contexts and have improved runtime performance.

## Conclusions

PACIFIC is a powerful end-to-end and easy to use tool that predicts the presence of SARS-CoV-2, Influenza, Metapneumovirus, Rhinovirus and other Coronavirus class-derived sequences directly from RNA-seq data with high sensitivity and specificity. PACIFIC will enable effective monitoring and tracking of viral infections and co-infections in the population in the context of the COVID-19 global pandemic and allow for the development of new strategies in molecular epidemiology of co-infections to understand variable host responses and improve the management of infectious diseases caused by viruses.

## Methods

PACIFIC and other associated software written for this manuscript is available at https://github.com/pacific-2020/pacific. We have used Python (version 3), scipy (v1.4.1), numpy (v1.18.1), scikit (v0.23.1), pandas (v1.0.1), tensorflow (v2.2.0), keras (v2.3.1), R (v3.6), tidyverse (v1.3.0), Biobase (v2.46.0) and Perl (v5.26) in our analysis.

### Training data

We downloaded 362 virus genomes from the NCBI assembly database corresponding to five classes of single stranded RNA viruses (Table 3, Supplementary Table S1). GenBank assembly identifiers and assembly versions with other metadata are listed in Supplementary Table S1. Since our focus was to detect co-infections with SARS-CoV-2, we made a separate class for SARS-CoV-2 containing 87 different assemblies (Table 3). The *Coronaviridae* class contained 12 genomes of alpha, beta, gamma and unclassified coronaviruses. The Influenza class contained assemblies of influenza A (H1N1, H2N2, H3N2, H5N1, H7N9, and H9N2 strains) and influenza B viruses. For the Rhinovirus class, assemblies of rhinovirus A (including A1 strain), B, C (including C1, C2, and C10 strains), and unlabelled enterovirus were grouped together. There were five distinct assemblies for metapneumovirus which were grouped into a single class. We included Human GENCODE^47^ canonical transcript sequences (downloaded from Ensembl v99 database^48^) as an additional class to distinguish sequencing reads derived from the human transcriptome. We generated between 0.44 and 3.5 million 150nt-long fragments in silico for each class using a custom Perl script available at https://github.com/pacific-2020/pacific (generatetestdata.pl, Table 3). These training sequences were randomly sampled without any base substitutions and were derived from both strands of the genome assemblies.

**Table 3:** Summary of training classes used for PACIFIC.

Class	Total reads	Number of genome assemblies	Number of taxonomic units	Included species/genus groups
Coronaviridae [ssRNA( +)]	644,483	12	12	Alpha, beta, gamma and unclassified coronaviruses
Influenza [ssRNA(−)]	1,073,237	128	125	Influenza A (H1N1, H2N2, H3N2, H5N1, H7N9, H9N2), Influenza B
Metapneumovirus [ssRNA(−)]	443,974	5	1	Metapneumovirus
Rhinovirus [ssRNA(+)]	1,339,435	130	107	Rhinovirus A and A1, B, C (C1, C2, C10), other enterovirus
SARS-CoV-2 [ssRNA(+)]	865,303	87	1	SARS-CoV-2
Human	3,531,425	1^a^	1	Human transcriptome
Total	7,897,857	363	247	

^a^GENCODE canonical transcripts were used to represent human reads in RNA-seq data.

### Model architecture

PACIFIC was implemented using the Keras API with a TensorFlow backend. Input reads were converted into 9-mers with a stride of 1, forming a vocabulary size of 4^9^ = 262,144 k-mers. Each of these k-mers is assigned a number using the Tokenize API from Keras^49^ from 1 to 262,144. The first index position of 0 is reserved to denote zero-padding for variable length sequences. Tokens are fed into the first hidden layer of the neural network and transformed into continuous vectors of length 100. After the embedding, a convolutional layer takes the previous numerical vectors and uses 128 convolution filters with a kernel size of 3. A pooling layer is used after the convolution, using *max pooling* with a kernel size of 3. A BiLSTM layer then follows, which uses two regular LSTMs; one starts ‘reading’ the input sequence from one of the two flanks, and the other from the opposite end. The output of the two LSTMs is then combined and passed to the next layer. Finally, PACIFIC has a fully connected layer using a *softmax* function to calculate posterior probabilities for each of the six classes. To reduce overfitting, we used 20% dropout at each hidden layer.

Cross-entropy was used as the loss function and ADAM^50^ was used as the optimizer. The final configuration of the network, hyperparameter tuning and the number and configurations of layers was obtained after several iterations between training and validation data. The final model is implemented *as double prediction* on both strands of the input sequence, whereby the forward and reverse-complement of the input sequence are predicted for class assignment. Classes for both predictions were required to match. The threshold of posterior probability for the assigned class was ≥ 0.95.

### PACIFIC training

NVIDIA GeForce RTX2080Ti was used to accelerate training. We trained two LSTM implementations, one using the fast LSTM implementation backed by CuDNN, supported only with NVIDIA Graphical Processing Unit (GPU). The other model was built using the regular implementation of LSTM. Both models achieved the same results. We started the training by shuffling the training sequences, using chunks of 200,000 reads to avoid loading all reads into memory. 90% of the data was used for training and 10% for optimization of parameters. After 15 chunks, the model converged on the validation set and training was halted. During training, we used categorical accuracy (1), binary accuracy (2) and cross-entropy loss from the optimization set to monitor the training.

1 $$Categorical \;accuracy = \frac{\# correct \;predictions}{{\# total \;predictions}}$$

2 $$Binary \;accuracy = \frac{\# correct \;predictions}{{\# total \;predictions}}\;{\text{if }}\;{\text{highest }}\;{\text{output }}\;{\text{probability }} > 0.{5}$$

Training was completed when the model converged, obtaining final categorical and binary accuracy values of 0.99, and 0.003 for optimization loss. PACIFIC model training was completed in 2.23 h on a machine with an AMD Ryzen Threadripper 2950 × 16-core processor, NVIDIA GeForce RTX2080Ti GPU, and 125.7 GiB RAM running Ubuntu 18.04.2 LTS.

To accommodate the inclusion of new classes and/or incorporate natural variation, we have designed a transfer learning strategy, which allows users to update the model with new data at reduced computational cost^51,52^. The transfer learning approach uses existing model parameters instead of random weights from a Gaussian distribution to train the model. We show applicability of this technique in extending the PACIFIC model to predict respiratory syncytial virus (RSV) sequences. Details of this extension are provided in Supplementary File 2.

### PACIFIC test datasets

We generated 100 independent test datasets using the ART sequence simulation software (version 2.5.8^30^) with default error models for substitutions, insertions and deletions using the Illumina HiSeq 2500 sequencing platform. For each dataset, we set seeds starting from 2021 to 2120 using a random number generator for reproducibility. Synthetic data contained 150nt single end reads derived from seven classes; the five model virus classes, a human class, and an “unrelated” class composed of 32,550 distinct virus genomes downloaded from the NCBI Assembly database. We sampled ~ 100,000 reads per class using a class-specific fold-coverage parameter to generate ~ 700,000 reads per test data (Table 4). Approximately 22% of reads contained mismatches, insertions or deletions relative to their respective reference sequences, reflecting error profiles of the Illumina sequencing platform. This process was automated using a custom script (generatebenchmarkdata.pl).

**Table 4:** Summary of benchmark datasets.

Sequence class	Total bases	Fold coverage	Number of reads	Reads with mismatches or indels
Coronaviridae	323,274	47.5	102,076	22,295–22,865
Human	75,434,059	0.227	100,208–100,209	21,768–22,425
Influenza	1,684,539	9.65	100,038–100,290	21,825–22,570
Metapneumovirus	66,596	228	100,548	21,890–22,559
Rhinovirus	925,412	16.7	101,940–101,953	22,196–22,919
SARS-CoV-2	2,599,395	5.8	100,337–100,349	21,831–22,507
Unrelated viruses	1,038,794,620	0.01738	100,175–100,196	21,890–22,479

### PACIFIC performance tests

PACIFIC was used to assign class labels to reads in the test data, and performance metrics were calculated by comparing known and predicted labels for each read. A read was assigned a class if the maximum posterior probability score for a class was ≥ 0.95. A true positive (TP) was defined when the true label and the predicted label were the same for a read. A true negative (TN) was defined when a read that did not belong to the true class was correctly predicted as a class different from the true class. False positives (FP) were reads which were predicted to be as the true class, although they originated from a different class. False negatives (FN) were all reads belonging to the true class but were predicted as a different class. An example confusion matrix for SARS-CoV-2 is described in Table 5. Precision, recall, accuracy, false positive rate and false negative rate were calculated [Eqs. (3–7)].

3 $$Precision = \frac{TP}{{TP + FP}}$$

4 $$Recall = \frac{TP}{{TP + FN}}$$

5 $$Accuracy = \frac{TP + TN}{{TP + TN + FP + FN}}$$

6 $$FPR = \frac{FP}{{FP + TN}}$$

7 $$FNR = \frac{FN}{{FN + TP}}$$

**Table 5:** Confusion matrix using SARS-CoV-2 as an example of a positive class.

	True/actual condition		
		Positive SARS-CoV-2+	Negative All other classes
*Predicted condition*	SARS-CoV-2+	True positive (TP)	False positive (FP)
	All other classes	False negative (FN)	True negative (TN)

where TP = True positive, FP = False positive, TN = True negative, FN = False negative, FPR = False positive rate, FNR = False negative rate.

### Establishing false positive rate thresholds for each class

This experiment was performed to quantify the impact of variable proportions of reads from each class on the percentage of false positives and to establish the detection threshold for each virus class in RNA-seq data. For each viral class in PACIFIC, we generated 100 datasets containing 500,000 reads derived from 4 out of the 5 viral classes, the human transcriptome and unrelated viral genomes in variable proportions. Reads were simulated using the ART software ^30^ with Illumina HiSeq 2500 error profiles that were 150nt long and modelled single end experiments. One of the five viral classes that was excluded was considered as the test class. This process was automated using a custom script (generatefprdata.pl). Subsequently, PACIFIC was run in *double prediction* to assign classes to each read. To calculate the percentage of false positives in each experiment, we counted the number of reads predicted as the absent test class and divided by the total number of reads.

### PACIFIC predictions in divergent SARS-CoV-2 sequences

To test PACIFIC’s ability to predict SARS-CoV-2 sequences with natural sequence variation, we downloaded 9,852 SARS-CoV-2 sequences from the GISAID database^53,54^, deposited between 1st October 2019 and 14th March 2020. We labelled this set with relatively lower natural variation as ‘low-divergence’. We also downloaded 12,667 SARS-CoV-2 sequences deposited between 1st October 2020 and 4th November 2020 and labelled them as ‘high-divergence’. All assemblies were aligned to the current SARS-CoV-2 reference genome (RefSeq ID: NC_045512.2) using BLASTN with default parameters to identify number of mismatches compared to the reference genome. To generate in silico reads for testing from these assemblies, we used a custom Perl script (generatetrainingdata.pl) to randomly sample ~ 100,000 in silico 150 bp reads 100 times from both low- and high divergence sets. PACIFIC was then independently run on all 200 datasets to assess prediction performance.

### Detecting viruses in human datasets and comparison with other tools

We downloaded 63 RNA-seq experiments from NCBI SRA database. Run accessions and other metadata details are supplied in Supplementary Table S2. All data were downloaded from the NCBI database using the SRA Toolkit *prefetch* and *fastq-dump* commands and applying the *–gzip* and *–fasta* options^55^. For the GALA II cohort study with 48 RNA-seq datasets and read lengths 18-390nt, we discarded reads < 150nt long. We then used PACIFIC to assign the presence/absence of each virus class in all 63 samples using the detection thresholds established in the previous section. We compared PACIFIC’s predictions with two alternative methods for virus detection: an alignment-based approach using BWA-MEM^56^, and a k-mer based approach using Kraken2^19^, described below.

For BWA-MEM^56^, all reads were mapped using default parameters to a combined reference containing assembly sequences for the five viral classes and the human transcriptome used for training PACIFIC (Table 3). Reads were assigned to a virus class based on the class membership of the genome assembly as described in Table 3 and Supplementary Table S1. For Kraken2, we first downloaded the Kraken taxonomy database and built a k-mer database using the same genomes used to train PACIFIC (Table 3). Kraken2 was then run using the –*use-names* flag, and output reads were parsed using species scientific names and reads were assigned a class based on the class membership of the genome assembly (Supplementary Table S1, Table 3). To fairly compare all three methods, we applied class detection thresholds as determined for and used in PACIFIC (Table 2) for the presence or absence of a virus class within a sample.

To investigate the origin of reads for all reads in samples that were discordantly predicted for the presence of a virus class by PACIFIC, BWA-MEM or Kraken2, we used the BLAST suite (v2.10.1+)^57,58^ to align reads to the NCBI nucleotide (*nt*) database, which includes sequences from all domains of life. We took the best hit from the pairwise alignment for each read, filtering for alignments with an E-value < 1e−6. BLASTN was used with the following parameters: *-task ‘megablast’ -max_target_seqs 1 -max_hsps 1 -evalue 1e-6* to query discordant viral class assignments between PACIFIC, BWA-MEM and Kraken2.

## Supplementary Information

Supplementary Information 1.

Supplementary Tables.

## Data Availability

Source code is available in the PACIFIC Github repository [https://github.com/pacific-2020/pacific/] under the MIT License. Publicly available data generated or analysed during this study are included in the following published articles^31,32,34^, accession numbers and other metadata are described in [https://github.com/pacific-2020/pacific/tree/master/metadata]. Other supplementary information and test data are available and can be downloaded from [https://cloudstor.aarnet.edu.au/plus/s/sRLwF3IJQ12pNGQ].

## References

[1] World Health Organization. WHO: The top 10 causes of death. *24 Maggio* 1–7 (2018). https://www.who.int/news-room/fact-sheets/detail/the-top-10-causes-of-death. Accessed 17 June 2020.

[2] Legand A (2013). Addressing the public health burden of respiratory viruses: The Battle against Respiratory Viruses (BRaVe) Initiative. Future Virol..

[3] Soriano JB (2020). Prevalence and attributable health burden of chronic respiratory diseases, 1990–2017: A systematic analysis for the Global Burden of Disease Study 2017. Lancet Respir. Med..

[4] Tang JW (2017). Global epidemiology of non-influenza RNA respiratory viruses: Data gaps and a growing need for surveillance. Lancet. Infect. Dis..

[5] Cui J, Li F, Shi ZL (2019). Origin and evolution of pathogenic coronaviruses. Nat. Rev. Microbiol..

[6] Al-Omari A, Rabaan AA, Salih S, Al-Tawfiq JA, Memish ZA (2019). MERS coronavirus outbreak: Implications for emerging viral infections. Diagn. Microbiol. Infect. Dis..

[7] Centers for Disease Control and Prevention (CDC) (2003). Revised US surveillance case definition for severe acute respiratory syndrome (SARS) and update on SARS cases–United States and worldwide December, 2003. MMWR Morb. Mortal Wkly. Rep..

[8] WHO EMRO|MERS situation update, January 2020|MERS-CoV|Epidemic and pandemic diseases. http://www.emro.who.int/pandemic-epidemic-diseases/mers-cov/mers-situation-update-january-2020.html. Accessed 13th July 2020.

[9] Bezerra PGM (2011). Viral and atypical bacterial detection in acute respiratory infection in children under five years. PLoS ONE.

[10] May L (2019). Rapid multiplex testing for upper respiratory pathogens in the emergency department: A randomized controlled trial. Open Forum Infect. Dis..

[11] Kim D, Quinn J, Pinsky B, Shah NH, Brown I (2020). Rates of Co-infection between SARS-CoV-2 and other respiratory pathogens. JAMA.

[12] Tong X (2020). Clinical characteristics and outcome of influenza virus infection among adults hospitalized with severe COVID-19: A retrospective cohort study from Wuhan, China. JAMA.

[13] Wang G (2020). Is co-infection with influenza virus a protective factor of COVID-19?. SSRN Electron. J..

[14] Rockett RJ (2020). Revealing COVID-19 transmission in Australia by SARS-CoV-2 genome sequencing and agent-based modeling. Nat. Med..

[15] Elnifro EM, Ashshi AM, Cooper RJ, Klapper PE (2000). Multiplex PCR: Optimization and application in diagnostic virology. Clin. Microbiol. Rev..

[16] Breitwieser FP, Lu J, Salzberg SL (2019). A review of methods and databases for metagenomic classification and assembly. Brief. Bioinform..

[17] Wood DE, Salzberg SL (2014). Kraken: Ultrafast metagenomic sequence classification using exact alignments. Genome Biol..

[18] Breitwieser FP, Baker DN, Salzberg SL (2018). KrakenUniq: Confident and fast metagenomics classification using unique k-mer counts. Genome Biol..

[19] Wood DE, Lu J, Langmead B (2019). Improved metagenomic analysis with Kraken 2. Genome Biol..

[20] Bzhalava Z, Tampuu A, Bała P, Vicente R, Dillner J (2018). Machine Learning for detection of viral sequences in human metagenomic datasets. BMC Bioinform..

[21] Liang Q, Bible PW, Liu Y, Zou B, Wei L (2020). DeepMicrobes: taxonomic classification for metagenomics with deep learning. NAR Genom. Bioinform..

[22] Tampuu A, Bzhalava Z, Dillner J, Vicente R (2019). ViraMiner: Deep learning on raw DNA sequences for identifying viral genomes in human samples. PLoS ONE.

[23] Li, H., Li, X., Caragea, D. & Caragea, C. Comparison of word embeddings and sentence encodings as generalized representations for crisis tweet classification tasks. *Proc. ISCRAM Asian Pacific 2018 Conf.* 1–13 (2018).

[24] Trabelsi A, Chaabane M, Ben-Hur A (2019). Comprehensive evaluation of deep learning architectures for prediction of DNA/RNA sequence binding specificities. Bioinformatics.

[25] Gong, Y., Wang, L., Guo, R. & Lazebnik, S. Multi-scale orderless pooling of deep convolutional activation features. in *Lecture Notes in Computer Science (including subseries Lecture Notes in Artificial Intelligence and Lecture Notes in Bioinformatics)* **8695 LNCS**, 392–407 (Springer Verlag, 2014).

[26] Siami-Namini, S., Tavakoli, N. & Namin, A. S. The performance of LSTM and BiLSTM in forecasting time series. in *2019 IEEE International Conference on Big Data (Big Data)* 3285–3292 (2019). 10.1109/BigData47090.2019.9005997

[27] Clark K, Karsch-Mizrachi I, Lipman DJ, Ostell J, Sayers EW (2016). GenBank. Nucleic Acids Res..

[28] Zhang Q, Jun SR, Leuze M, Ussery D, Nookaew I (2017). Viral phylogenomics using an alignment-free method: A three-step approach to determine optimal length of k-mer. Sci. Rep..

[29] Lin J (1991). Divergence measures based on the Shannon entropy. IEEE Trans. Inf. Theory.

[30] Huang W, Li L, Myers JR, Marth GT (2011). ART: a next-generation sequencing read simulator. Bioinformatics.

[31] Blanco-Melo D (2020). Imbalanced host response to SARS-CoV-2 drives development of COVID-19. Cell.

[32] Wu F (2020). A new coronavirus associated with human respiratory disease in China. Nature.

[33] Kumar R (2013). Factors associated with degree of atopy in Latino children in a nationwide pediatric sample: The Genes-environments and Admixture in Latino Asthmatics (GALA II) study. J. Allergy Clin. Immunol..

[34] Wesolowska-Andersen A (2017). Dual RNA-seq reveals viral infections in asthmatic children without respiratory illness which are associated with changes in the airway transcriptome. Genome Biol..

[35] Wesolowska-Andersen A (2018). Correction: Dual RNA-seq reveals viral infections in asthmatic children without respiratory illness which are associated with changes in the airway transcriptome [Genome Biol., 18, (2017) (12)]. 10.1186/s13059-016-1140-8. Genome Biol..

[36] Tapparel C (2007). New complete genome sequences of human rhinoviruses shed light on their phylogeny and genomic features. BMC Genom..

[37] Tapparel C (2009). New respiratory enterovirus and recombinant rhinoviruses among circulating picornaviruses. Emerg. Infect. Dis..

[38] Aynaud MM (2020). Transcriptional programs define intratumoral heterogeneity of ewing sarcoma at single-cell resolution. Cell Rep..

[39] Wynants L (2020). Prediction models for diagnosis and prognosis of covid-19 infection: Systematic review and critical appraisal. BMJ.

[40] WHO (2013). Research needs for the battle against respiratory viruses (BRaVe). Future Virol..

[41] Langelier C (2018). Integrating host response and unbiased microbe detection for lower respiratory tract infection diagnosis in critically ill adults. Proc. Natl. Acad. Sci. USA.

[42] Graf EH (2016). Unbiased detection of respiratory viruses by use of RNA sequencing-based metagenomics: A systematic comparison to a commercial PCR panel. J. Clin. Microbiol..

[43] Brinkmann A (2019). Proficiency testing of virus diagnostics based on bioinformatics analysis of simulated in silico high-throughput sequencing data sets. J. Clin. Microbiol..

[44] Hogan CA, Sahoo MK, Pinsky BA (2020). sample pooling as a strategy to detect community transmission of SARS-CoV-2. JAMA.

[45] Sanjuán R, Nebot MR, Chirico N, Mansky LM, Belshaw R (2010). Viral mutation rates. J. Virol..

[46] Sanjuán R, Domingo-Calap P (2016). Mechanisms of viral mutation. Cell. Mol. Life Sci..

[47] Frankish A (2018). GENCODE reference annotation for the human and mouse genomes. Nucleic Acids Res..

[48] Yates AD (2019). Ensembl 2020. Nucleic Acids Res..

[49] Keras Team. Keras: Deep learning for humans. *GitHub repository*. https://github.com/keras-team/keras (2020).

[50] Kingma, D. P. & Ba, J. L. Adam: A method for stochastic optimization. in *3rd International Conference on Learning Representations, ICLR 2015: Conference Track Proceedings* (International Conference on Learning Representations, ICLR, 2015).

[51] Weiss K, Khoshgoftaar TM, Wang DD (2016). A survey of transfer learning. J. Big Data.

[52] Torrey, L. & Shavlik, J. Transfer Learning. in *Handbook of Research on Machine Learning Applications* (ed. Soria, E., Martin, J., Magdalena, R., Martinez, M., Serrano, A.) (IGI Global, 2009).

[53] Shu Y, McCauley J (2017). GISAID: Global initiative on sharing all influenza data: from vision to reality. Eurosurveillance.

[54] Elbe S, Buckland-Merrett G (2017). Data, disease and diplomacy: GISAID’s innovative contribution to global health. Glob. Challenges.

[55] SRA Toolkit Development Team (2018). SRA. Toolkit..

[56] Li, H. Aligning sequence reads, clone sequences and assembly contigs with BWA-MEM. (2013). https://arxiv.org/abs/1303.3997.

[57] Altschul SF, Gish W, Miller W, Myers EW, Lipman DJ (1990). Basic local alignment search tool. J. Mol. Biol..

[58] Morgulis A (2008). Database indexing for production MegaBLAST searches. Bioinformatics.

