# Supplementary material for: PACIFIC: a lightweight deep-learning classifier of SARS-CoV-2 and co-infecting RNA viruses
Source: Sci Rep. 2021 Feb 5;11:3209. doi: 10.1038/s41598-021-82043-4 (PMC7864945; doi:10.1038/s41598-021-82043-4)
Supplement: Supplementary file 1 — Supplementary Information 1. [file 41598_2021_82043_MOESM1_ESM.docx]

# **PACIFIC: A lightweight deep-learning classifier of SARS-CoV-2 and co-infecting RNA viruses**

Pablo Acera Mateos^1,2†^, Renzo F. Balboa^1,3†^, Simon Easteal^1,3^, Eduardo Eyras^1,2,4,5*^, and Hardip R. Patel^1,3*^

^1^John Curtin School of Medical Research, Australian National University, Canberra, Australian Capital Territory 2600, Australia.

^2^EMBL Australia Partner Laboratory Network at the Australian National University, Canberra, Australian Capital Territory 2601, Australia.

^3^National Centre for Indigenous Genomics, Australian National University, Canberra, Australian Capital Territory 2600, Australia.

^4^IMIM - Hospital del Mar Medical Research Institute. E08003 Barcelona, Spain.

^5^Catalan Institution for Research and Advanced Studies. E08010 Barcelona, Spain.

Email addresses: [pablo.aceramateos@anu.edu.au](mailto:pablo.aceramateos@anu.edu.au) (PAM), [renzo.balboa@anu.edu.au](mailto:renzo.balboa@anu.edu.au) (RFB), [simon.easteal@anu.edu.au](mailto:simon.easteal@anu.edu.au) (SE), [eduardo.eyras@anu.edu.au](mailto:eduardo.eyras@anu.edu.au) (EE), [hardip.patel@anu.edu.au](mailto:hardip.patel@anu.edu.au) (HRP).

^†^These authors contributed equally to this work

*Correspondence: Hardip R. Patel ([hardip.patel@anu.edu.au](mailto:hardip.patel@anu.edu.au)) and Eduardo Eyras ([eduardo.eyras@anu.edu.au](mailto:eduardo.eyras@anu.edu.au))


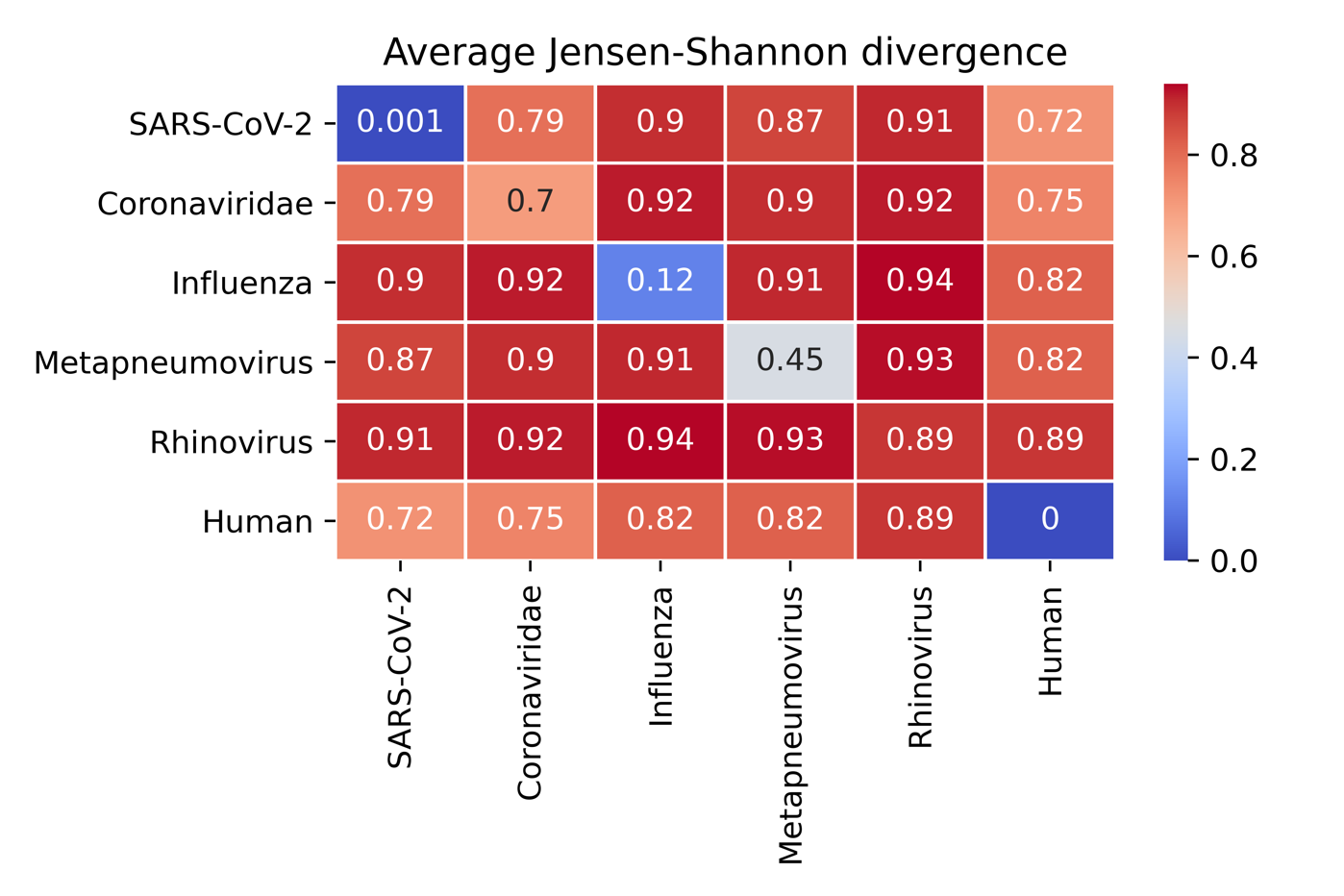
**Supplementary Figure S1. Inter/intra-class Jensen-Shannon Divergence (JSD).** All-vs-all JSD were calculated using 9-mers and averages calculated at a class level. Pair of identical pairs have a JSD of 0 for the pair (blue), while sequences that do not share any k-mers have a JSD value of 1 (red). Intra-class JSD were overall lower compared to the inter-class JSD as expected.


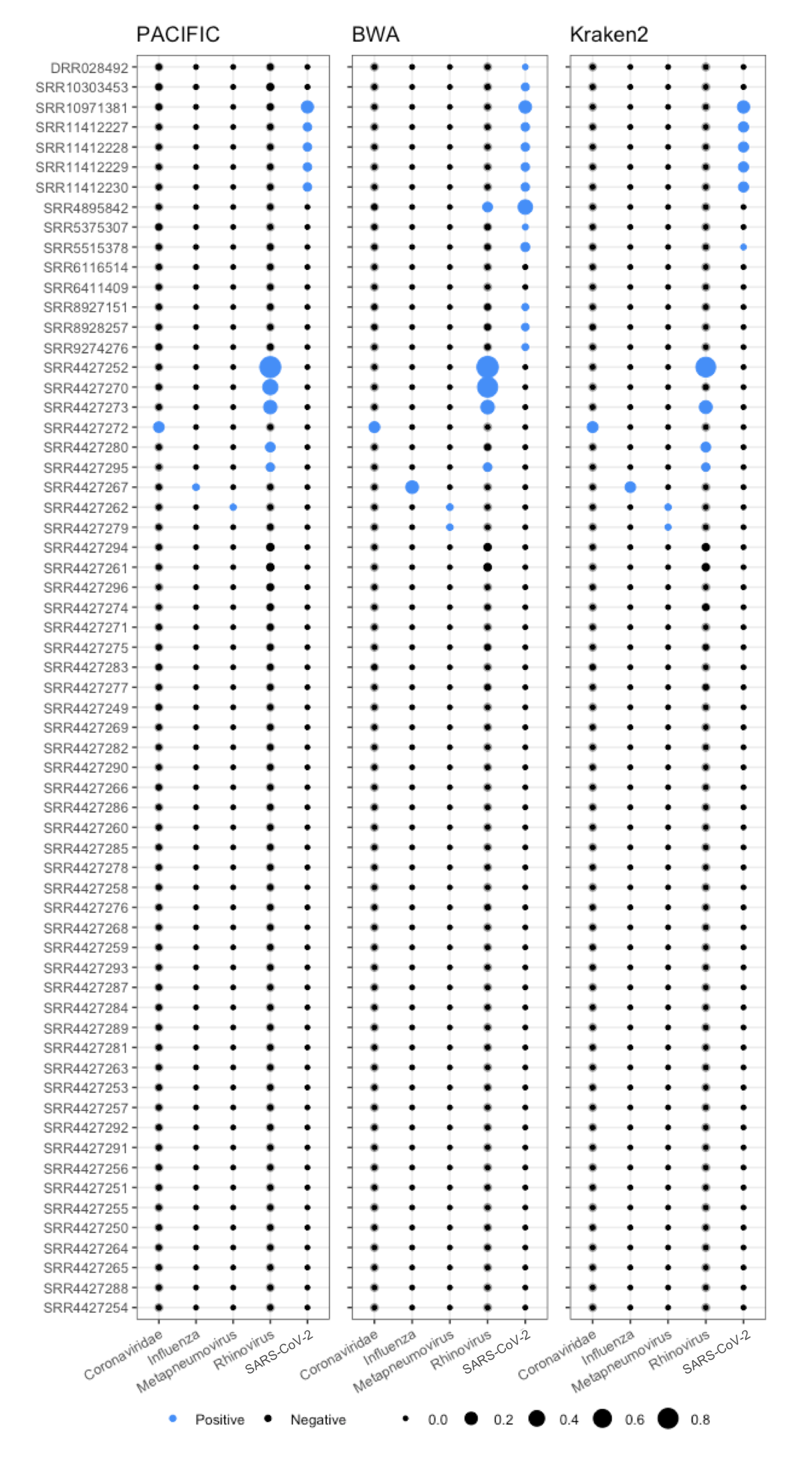


**Supplementary Figure S2. PACIFIC, BWA-MEM and Kraken2 predictions on all samples from all datasets used in this study ^31,32,34)^.** Spheres represent the percentage of predicted reads above 0.95 posterior probability score for PACIFIC (left), and the proportion of predicted viral reads using BWA-MEM (centre) and Kraken2 (right). Black circles represent predictions where the percentage of reads does not reach PACIFIC thresholds (Table 2). Blue circles represent samples predicted above PACIFIC class thresholds.

**Extension of BLAST analysis**

One sample (SRR4427279) was classified as being positive for the Metapneumovirus class by BWA-MEM and Kraken2 but not by PACIFIC. Best hits for all 28 reads aligned to human respiratory syncytial virus A (see Results). To further test whether these reads may be derived from metapneumovirus, we investigated secondary BLAST hits for these reads. We used BLASTN to align all 28 reads to the NCBI nucleotide *(nt)* database, taking the best hsp from pairwise alignments for a maximum of 10 target sequences for each read, using the parameters --*max_target_seqs 10 –*max_hsps *1*. Such searches revealed that all 28 reads had their best hits for their top 10 alignments to either human respiratory syncytial virus A sequences (267/280 pairwise alignments) or its genus Human orthopneumovirus (13/280 pairwise alignments); no hits to metapneumovirus sequences were found.

BWA-MEM, but not PACIFIC or Kraken2 also reported reads that aligned to Rhinovirus forone sample, with best hits from BLAST showing alignments primarily to *Homo sapiens* and *Pan paniscus* (SRR4895842; see Results). A similar strategy was used to test whether secondary hits from these reads were derived from rhinovirus using BLAST. We collected reads classified as Rhinovirus by BWA-MEM and used BLASTN against (*nt*) database to investigate the 10 best database matches per read. The majority of these hits belong to *Homo sapiens*, followed by *Pan paniscus.* In total, counting best and secondary hits there were 52 taxonomy IDs matching to these reads; none of these IDs corresponded to Rhinovirus, or any of the other model virus classes.

**Transfer learning allows for a lightweight solution to include new species of interest in PACIFIC**

To be able to predict new virus sequences not currently in PACIFIC, we aimed to provide a solution that updates the existing model to include new viral sequence information. Transfer learning takes a pre-trained model developed for a specific task and retrain or fine-tune that model to solve a related task, usually reducing computational cost and the number of inputs^52^ to converge. To test this approach, we aimed to integrate sequence information from 70 respiratory syncytial virus (RSV) assemblies downloaded from the NCBI Assembly database into PACIFIC as a new ‘RSV’ class. We generated reads from these assemblies using a custom Perl script available at <https://github.com/pacific-2020/pacific> (generatetestdata.pl), using 100x coverage for all RSV genomes, and added these reads to the original dataset generated using the same script. This new training dataset was then used to compare the transfer learning approach and the traditional approach of retraining the entire model. Our transfer learning approach initialises the weights using parameters derived from the previous model, then updates all layer parameters as in traditional training. We observed that the transfer learning approach converges faster than traditional training. Both loss and accuracy from the validation datasets improved considerably when comparing traditional training with transfer learning, reducing from 0.0467 to 0.0040 (~11.6-fold decrease) for the validation loss and increasing validation accuracy from 0.9858 to 0.9989 after one epoch respectively. We provide a script (transfer_learning_pacific.py) for the end-user that can be used to perform this operation.

To evaluate the performance of the transfer learning approach described above, we simulated 10 independent Illumina experiments using ART^30^. In each experiment, ~100, 000 *in silico* reads were generated per class, including RSV. We then used PACIFIC with and without the new ‘RSV’ class, to classify reads within each experiment, and evaluated FPR and FNR rates before and after applying the transfer learning approach. Overall, the model trained using transfer learning exhibited decreased FPRs and increased FNRs (Supplementary Fig. S3). Specifically, Coronaviridae and Rhinovirus classes had the highest decrease in false positives (a 20-fold and 45-fold decrease respectively). It is likely that RSV-derived reads were originally misclassified as Coronaviridae or Rhinovirus and are now correctly classified as part of the RSV class. However, we also observe an increase in false negatives using the new model, with Metapneumovirus and Coronavirus two classes exhibiting the largest increase in FNR (4 and 7.3 times respectively). In addition, the RSV class exhibits low FPRs and FNRs (~0.00000456 and ~0.00262 respectively). Overall, the new model shows increased precision at the cost of a decrease in recall.


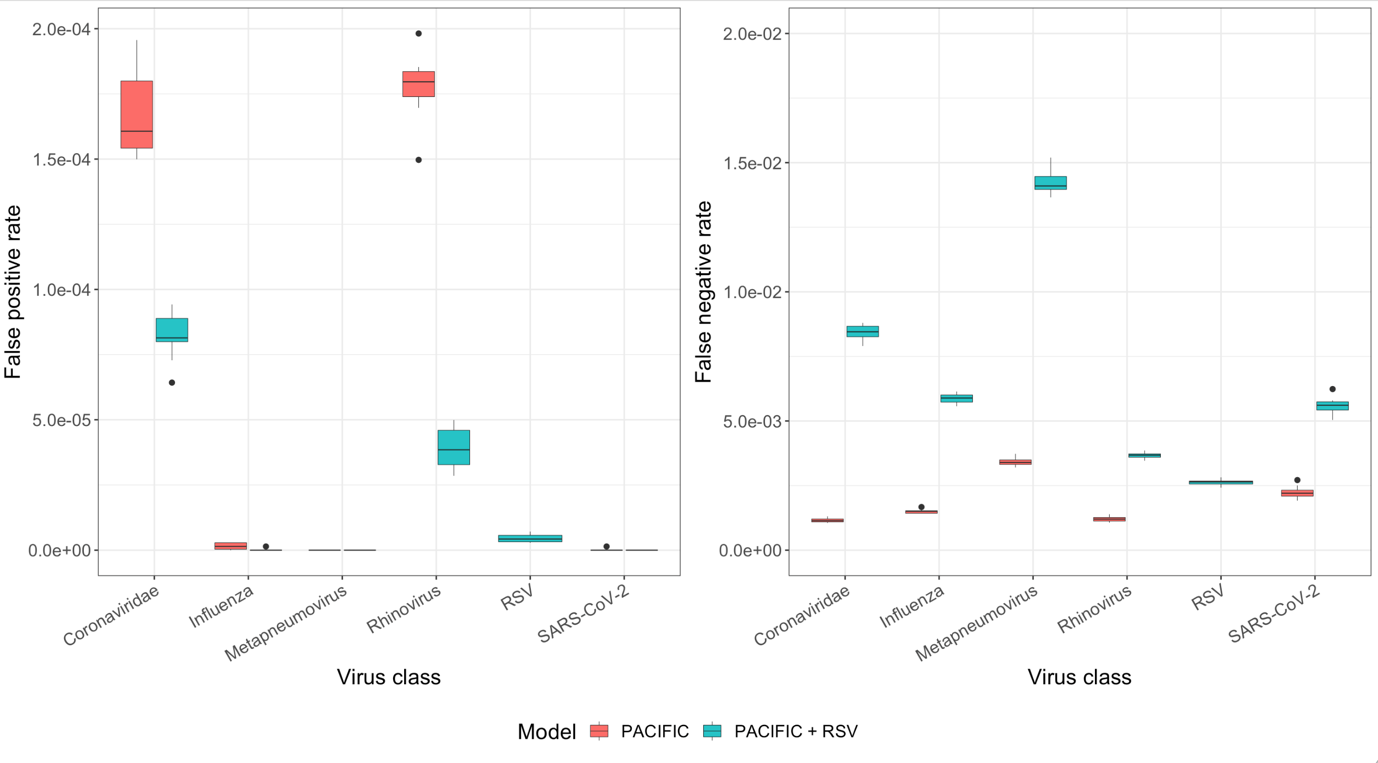


**Supplementary Figure S3.** False positive (left panel) and false negative (right panel) rates for reads predicted using the original model (PACIFIC, red), and for reads predicted after incorporating the respiratory syncytial virus (RSV) class into PACIFIC model (PACIFIC + RSV, green).
